# Supplementary material for: Mitochondrial levels determine variability in cell death by modulating apoptotic gene expression
Source: Nat Commun. 2018 Jan 26;9:389. doi: 10.1038/s41467-017-02787-4 (PMC5785974; doi:10.1038/s41467-017-02787-4)
Supplement: Supplementary file 1 — Supplementary Information [file 41467_2017_2787_MOESM1_ESM.pdf]

SUPPLEMENTARY METHODS:

MATHEMATICAL MODEL. RELATED TO EXPERIMENTAL PROCEDURES.

We based our computer simulations of TRAIL-induced apoptosis on previous kinetic models developed for the apoptotic extrinsic pathway (Fig. 1a)[1, 2, 3, 4, 5]. Briefly, these Extrinsic Apoptosis Reaction Models (EARM) use the mass action law for the reactions involved in the apoptotic route, to write down a system of ordinary differential equations (ODEs) giving the time evolution of all protein species considered (Supplementary Table 1). The kinetic parameters (reaction rates and initial protein numbers) have been fitted and improved to reproduce a wide range of experimental data including pathway RNAi and protein overexpression[2], variability in apoptosis times[1, 4], different apoptotic phenotypes[6, 7] or fractional killing[5].

The goal of this work is to understand how mitochondrial variability influences apoptosis, and thus we will explicitly introduce the effect of the initial amount of mitochondrial mass in the EARM taking into account our own experimental observations. Previous work has shown that variability in times to death is a consequence of differences in the abundance of specific proteins involved in the apoptotic pathway[3, 4]. Our experiments show that cell-to-cell variability of the apoptotic proteins is correlated with variability in mitochondrial levels (Fig. 3b and c). We will use these correlations to constrain sampling of initial protein levels used as input in the EARM, as explained in the next sections. Cell-to-cell heterogeneity in mitochondrial mass is also included in the model by sampling the initial values of the variable *Pore* (Supplementary Table 1), according to our experimental distribution of CMXRos levels in a HeLa clonal population. In the EARM model, the variable *Pore* represents the number of potential sites in the mitochondrial membrane where a pore could be formed. When a Bax tetramer binds to the mitochondrial membrane the variable *Pore* turns into an activated variable denoted as *Pore** (reaction #19 in Supplementary Table 2) that actually represents a mitochondrial pore able to release *Cytochrome-C* and *Smac* to the cytosol (reactions #20 and #21 in Supplementary Table 2). As in previous versions of EARM[2, 3], the number of potential binding sites for Bax are in excess and thus heterogeneity in the variable *Pore* will play a minor role.

The biochemical reactions in the model follow the generic structure (see Supplementary Table 2):

$$A + B \underset{k_r^{(i)}}{\overset{k^{(i)}}{\rightleftarrows}} C \overset{k_c^{(i)}}{\longrightarrow} D + E$$

with i being the index for the i-th reaction and with specific forward (k), reverse (k_r) and catalytic (k_c) rates for each reaction. All rates have been taken from[3] and rescaled by a global factor of 6, with the only exception of $k^{(1)}$ that was tuned manually to best fit our experimental data.

Besides the biochemical reactions given in Supplementary Table 2, the model includes synthesis and degradation of the proteins involved. The synthesis rate (k_s) for the i-th protein is set to:

$$k_s^{(i)} = k_{deg}^{(i)} \times X^{(i)}$$

with X the initial number of molecules of that protein (which depends on the mitochondrial content of the cell as explained in the next section), and k_{deg} its degradation rate. Degradation rates are given in Supplementary Table 1.

Initializing protein levels constrained by mitochondrial abundance.

First, we found that distributions of both mitochondrial mass and protein levels in a population of HeLa cells are well represented by a log-normal distribution (both Lilliefors test and χ^2 -test for goodness of fit to a log-normal agreed with a log-normal distribution of protein and mitochondrial levels to 5% significance). Using this distribution, we can sample protein abundances constrained by mitochondrial levels as follows: denote by m and p the amounts of mitochondria and protein in a certain cell, normalized by the population respective means. We define a set of transformed variables as

$$x \equiv \log(m)$$
$$y \equiv \log(p) \tag{S1}$$

Since m and p are log-normally distributed, both x and y will follow a normal distribution. Knowing the mean of x and y (μ_x, μ_y), their standard deviations (σ_x, σ_y), as well as their Pearson's correlation coefficient (ρ), the probability of finding a cell with a particular value of x and y would then be given by a bivariate normal distribution, $P(x,y)$.

$$P(x,y) = \frac{1}{2\pi \cdot \sigma_x \cdot \sigma_y \cdot \sqrt{1-\rho^2}} \times \exp\left[\frac{-z}{2 \cdot (1-\rho^2)}\right]$$
$$z \equiv \left(\frac{x-\mu_x}{\sigma_x}\right)^2 - 2\rho\left(\frac{x-\mu_x}{\sigma_x}\right)\left(\frac{y-\mu_y}{\sigma_y}\right) + \left(\frac{y-\mu_y}{\sigma_y}\right)^2 \tag{S2}$$

The expression for $P(x,y)$ in (S2) can be re-written as:

$$P(x,y) = \frac{1}{\sqrt{2\pi}\sigma_x} \cdot e^{-(x-\mu_x)^2 / 2\sigma_x^2} \times \frac{1}{\sqrt{2\pi}\sigma_{yi}} \cdot e^{-(y-\mu_{yi})^2 / 2\sigma_{yi}^2}$$
$$\mu_{yi} \equiv \mu_y + \rho\frac{\sigma_y}{\sigma_x}(x-\mu_x) \tag{S3}$$
$$\sigma_{yi} \equiv \sigma_y \cdot \sqrt{1-\rho^2}$$

The expression in (S3) can be identified as the probability of obtaining a value x when sampling from a normal distribution ($P(x)$, in green), multiplied by the conditional probability of obtaining a value y once that x has been fixed ($P(y|x)$, in red), that follows a normal distribution with new mean μ_{yi} (which depends on the value of x set) and standard deviation σ_{yi} as defined above.

We then proceed in the following way:

1) We get a value for x sampling randomly from a normal distribution with mean and standard deviation obtained from the fitted experimental values of CMXRos, and m as $m = \exp(x)$, (S1).
2) With this value for x and the values of μ_y, σ_y and ρ for each protein (obtained from the immunolabelling experiments), we can calculate μ_{yi} and σ_{yi} as seen in (S3).
3) We sample y from a normal distribution with mean μ_{yi} and standard deviation σ_{yi}.
4) We calculate p as $p = \exp(y)$, see (S1). We do this for all the protein species included in the model.
5) We obtain protein abundances in absolute number of molecules (see below and Supplementary Table 1), which we use as input for the EARM. With this we numerically solve the model equations to obtain fate and death time (see next section) for each simulated cell.

Note that we started working with m and p, which are experimentally given in arbitrary units of fluorescence intensity, and later normalized by the average intensity of the population (mean 1). The EARM requires that we set initial values for the number of molecules of proteins and mitochondria. To match this requirement, and similar to previous analysis with EARM[3, 4], we provide population average numbers for each protein type as parameters. These average copy numbers, together with the values of the reaction rates, can be tuned within biologically plausible ranges to reproduce the experimental results. Parameter values can be found in Supplementary Tables 1 and 2.

In summary, from our immunolabeling experiments we first calculate μ_x, μ_y, σ_x, σ_y and ρ for each protein. From the properties of the log-normal distribution, the values of μ_x, μ_y, σ_x and σ_y can be obtained as

$$\sigma_x = \sqrt{\log(1 + CV_m^2)}$$

$$\mu_x = \log \mu_m - \sigma_x^2/2$$

$$\sigma_y = \sqrt{\log\left(1 + CV_p^2\right)} \qquad \text{(S4)}$$

$$\mu_y = \log \mu_p - \sigma_y^2/2$$

knowing the means (μ_m and μ_p) and coefficients of variation (CV_m and CV_p) of the mitochondria and protein distributions respectively. The value of ρ is obtained by calculating the correlation between log(m) and log(p). Note that for the normalized data ($\mu_m = \mu_p = 1$), expressions (S4) simplify to $\sigma_x = \sqrt{\log(1 + \sigma_m^2)}$, $\mu_x = -\sigma_x^2/2$ and $\sigma_y = \sqrt{\log\left(1 + \sigma_p^2\right)}$, $\mu_y = -\sigma_y^2/2$. With these values of the distribution parameters we sample mitochondrial and protein levels from the joint distribution specified in (S3).

We will call this modelling approach mitoEARM (EARM with the addition of mitochondrial constraints on protein abundances) to distinguish it from the original EARM. Note that while in the previous approach by Spencer et al. initial protein levels are sampled either independently from experimental distributions[4], or taking into account several protein-protein correlations[3], here we sample protein levels conditioned to initial mitochondrial amount, using only the mitochondria-protein correlation and the variances of mitochondria and protein distributions. In fact, protein-protein correlations as observed by Gaudet et al.[3] arise naturally if a common source (mitochondrial mass) changes simultaneously the levels of both proteins (see section *Mitochondria-protein correlations constrain protein-protein correlations*).

Determining cell fate and times to death from model simulations.

The set of differential equations that build up the mitoEARM are numerically solved (Matlab2015a, Mathworks) to extract the temporal dynamics of the proteins involved in the pathway. From these dynamics, we need to impose a criterion to determine whether a cell will survive TRAIL treatment or not, and in the latter case, when will death happen.

It is known that most of the variability in apoptosis times comes from cells reaching MOMP at very different moments, while the timespan between MOMP and death is less variable[8]. Therefore, and similarly to the criterion used in previous modelling studies[3], we choose a high enough level of cytosolic Smac as the marker of time to death. In the mitoEARM we consider the time at which Smac reaches 90% of its maximum value as the cell's time to death.

Since the original EARM was developed to study variability in apoptosis times[3, 4], no restriction was imposed in the model to allow for survival of cells to drug addition, even at large doses and very long times after treatment. Model equations are deterministic and eventually, even for low initial levels of pro-apoptotic proteins, enough Smac will be released to consider any cell as death at long times. To mimic our experimental observation that a noticeable fraction of TRAIL-treated cells survive for longer than 24 h even at a saturating dose of the drug, we need to impose a criterion to decide cell fate within our modelling framework. A recent study has shown that cell fate is linked to Casp8 activation rate in HeLa cells treated with TRAIL and other pro-apoptotic compounds[9], so in the mitoEARM we focus on Casp8 dynamics to determine cell fate. Cells that have activated Casp8 at a rate greater than a threshold at some point during the first 24 h of treatment with TRAIL are counted as dying cells, while cells that don't reach the threshold are counted as survivors. The threshold is selected such that the model mimics the surviving fraction at a sensitive dose (32 ng ml^{-1}) of TRAIL treatment, and it is kept constant among TRAIL doses. This reflects the experimental finding that for a given cell line and drug, the Casp8 activation threshold that best separates survivors from apoptotic cell is dose invariant[9]. Increasing or decreasing TRAIL raises or lowers Casp8 activation rates, thus forcing a higher or lower fraction of cells to overcome the threshold.

With these criteria, we can determine whether a simulated cell survives or dies and, in the latter case, the time elapsed to time to death after TRAIL addition.

Parameter estimation and model calibration.

We started the model calibration with the original parameters (average protein copy numbers and kinetic rates) of the original EARM1.3 model[3]. For degradation rates, we use the values provided in Bertaux et al.[5]. To reproduce the range of experimental times to death, we rescaled all kinetic parameters by a common factor. Note that since all kinetic parameters appear as linear terms in the model equations, this is equivalent to rescale time.

To adjust the Casp8 activation rate to recover the experimental fraction of surviving cells at 32 ng ml^{-1}, we varied the binding constant of TRAIL ligand to DR5 receptor (Supplementary Table 1), as well as the average Casp8 levels, average receptor levels and the number of average TRAIL molecules per cell corresponding to a reference dose (Supplementary Table 2). Manual calibration of these few parameters was enough to qualitatively reproduce all our experimental observations (dose-response curve, variability in apoptosis times and discrimination of cell fate and times to death by mitochondrial content), as shown in Fig. 5.

Sensitivity analysis of mitochondrial-protein correlations.

The discrimination performance of mitochondrial mass as a cell fate classifier depends on its correlation with all the proteins in the apoptotic route. To test whether mitochondria is a good classifier as result of controlling the amount of some specific proteins along the route, we performed a sensitivity analysis of every node in the pathway with respect to its degree of correlation with mitochondria. By analogy with the local sensitivity analysis of kinetic models with respect to parameter variations[10], we define a local sensitivity coefficient for each protein i as

$$s_i^{AUC} = \frac{\rho_i^{exp}}{AUC^{exp}} \frac{\partial\, AUC}{\partial\, \rho_i}\bigg|_{\rho_i = \rho_i^{exp}} \qquad (S5)$$

where ρ_i is the logarithmic correlation of mitochondria with protein i, and superindex exp indicated magnitudes at their experimentally measured values. To calculate numerically the partial derivative, we use a finite difference approximation changing the correlations ρ_i around the experimental value, resampling new protein levels from (S3) and calculating the new AUC. The correlations for the rest of the proteins $\rho_{j \neq i}$ are kept at their experimental values.

Mitochondria-protein correlations constrain protein-protein correlations.

Given the mitochondrial mass (m) and the abundances of any two different protein species (p_1 and p_2) for each cell in a population, it is possible to prove that the correlation of each protein with mitochondria introduces constraints on the correlation between both of them. Let's start defining:

$$\begin{aligned} x &\equiv \log(m) \\ y &\equiv \log(p_1) \\ z &\equiv \log(p_2) \end{aligned} \qquad (S6)$$

Since m, p_1 and p_2 are log-normally distributed, x, y and z will follow normal distributions with respective means μ_x, μ_y and μ_z and standard deviations σ_x, σ_y and σ_z. We can define the following vectors:

$$\begin{aligned} \vec{v} &\equiv [x \quad y \quad z] \\ \vec{\mu} &\equiv [\mu_x \quad \mu_y \quad \mu_z] \end{aligned} \qquad (S7)$$

And the following covariance matrix:

$$\Sigma \equiv \begin{bmatrix} \sigma_x^2 & \sigma_{xy} & \sigma_{xz} \\ \sigma_{xy} & \sigma_y^2 & \sigma_{yz} \\ \sigma_{xz} & \sigma_{yz} & \sigma_z^2 \end{bmatrix} \tag{S8}$$

Where σ_{ij} denotes the covariance of variables i and j (with i=x,y,z and j=x,y,z). Since all our variables are normally distributed, it is true that:

$$\sigma_{ij} = \rho_{ij} \cdot \sigma_i \cdot \sigma_j \tag{S9}$$

being ρ_{ij} the Pearson correlation between the variables i and j. The multi-variate normal probability density function (PDF) describing any number of variables n is given by:

$$P(\vec{v}) = \frac{1}{(2\pi)^{n/2} \, |\, \Sigma \,|^{1/2}} \exp\left(-\frac{1}{2} (\vec{v} - \vec{\mu})^T \; \Sigma^{-1} \, (\vec{v} - \vec{\mu}) \right) \tag{S10}$$

In the general case, $\vec{v}$ and $\vec{\mu}$ are n-dimensional vectors and Σ a $n\times n$ covariance matrix of determinant $|\Sigma|$. In our example we have n=3, therefore the vectors take the forms in (S7) and the covariance matrix is the one in (S8). In order for (S10) to hold true, the covariance matrix Σ must be positive-definite, all of its first minors being positive. For its 2×2 minors, it's easy to see, using (S9), that:

$$\begin{vmatrix} \sigma_i^2 & \sigma_{ij} \\ \sigma_{ij} & \sigma_j^2 \end{vmatrix} = \sigma_i^2 \sigma_j^2 - \sigma_{ij}^2 = \sigma_i^2 \sigma_j^2 - \rho_{ij}^2 \sigma_i^2 \sigma_j^2 = \sigma_i^2 \sigma_j^2 \left(1 - \rho_{ij}^2\right) \tag{S11}$$

The expression (S11) is always greater than zero if not in the trivial situation of ρ_{ij}=1. As for the 3×3 determinant, again using (S9) we can obtain:

$$\begin{aligned} |\,\Sigma\,| &= \left(\sigma_x \sigma_y \sigma_z\right)^2 + 2\,\sigma_{xy}\sigma_{xz}\sigma_{yz} - \sigma_x^2 \sigma_{yz}^2 - \sigma_y^2 \sigma_{xz}^2 - \sigma_z^2 \sigma_{xy}^2 \\ &= \sigma_x^2 \sigma_y^2 \sigma_z^2 \times \left[1 + 2\,\rho_{xy}\rho_{xz}\rho_{yz} - \left(\rho_{xy}^2 + \rho_{xz}^2 + \rho_{yz}^2\right)\right] \end{aligned} \tag{S12}$$

The zeros of the expression (S12) are at:

$$\begin{aligned} \rho_{yz}^{(0+)} &= \rho_{xy}\rho_{xz} + \sqrt{\left(1 + \rho_{xy}\rho_{xz}\right)^2 - \left(\rho_{xy} + \rho_{xz}\right)^2} \\ \rho_{yz}^{(0-)} &= \rho_{xy}\rho_{xz} - \sqrt{\left(1 + \rho_{xy}\rho_{xz}\right)^2 - \left(\rho_{xy} + \rho_{xz}\right)^2} \end{aligned} \tag{S13}$$

It can be proven that any value for ρ_{yz} that falls between $\rho^{(0+)}$ and $\rho^{(0-)}$ will cause $|\Sigma|$ to be positive, while any other will make it negative. The maximum value for $|\Sigma|$ is reached in the middle of this interval:

$$\rho_{yz}^{(1/2)} = \rho_{xy}\rho_{xz} \tag{S14}$$

In order to build a consistent multivariate PDF, we need to satisfy the following condition for ρ_{yz}:

$$\rho_{yz}^{(0-)} < \rho_{yz} < \rho_{yz}^{(0+)} \tag{S15}$$

Remember that y and z are the log-transformed variables representing the amounts of any given pair of proteins inside a cell, being ρ_{yz} the log-correlation between them. This log-correlation is constrained between two values that depend on the log-correlation of each respective protein with the third variable, x (the log-transformed mitochondrial mass of the cell), namely ρ_{xy} and ρ_{xz}, as seen in (S13) and (S15).

Simply put, mitochondria-protein correlations restrict the available range for the corresponding protein-protein correlations. If there are no additional regulations between the two proteins, their 'basal' log-correlation will be ρ_{yz}=ρ_{xy} ρ_{xz} (S14). Extra layers of regulation can bring this value up or down, however keeping it within the permitted ranges (S15). The expected protein-protein correlation ranges taking into account the experimentally measured mitochondria-protein correlations, as well as their 'basal' correlation values (no other variables or interactions affect these correlations) are shown in Supplementary Table 3.

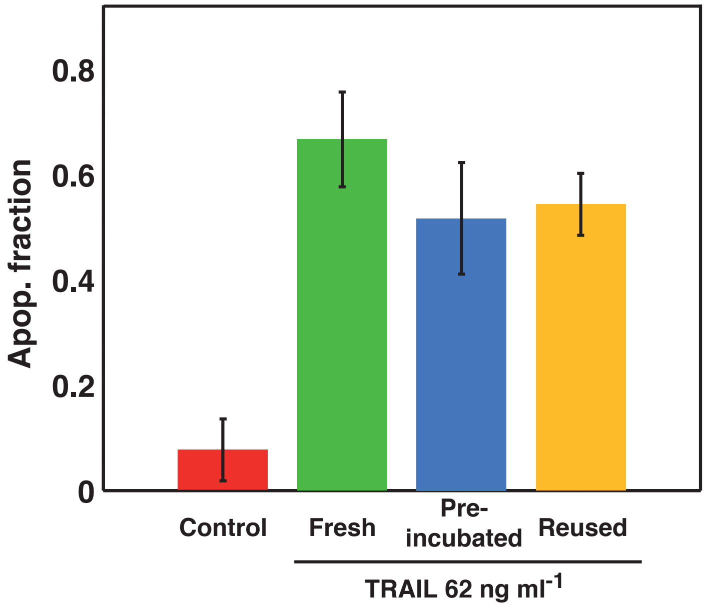

Supplementary Fig. 1. Stability of TRAIL during the experimental procedure. HeLa cells stained with MG were not treated (Control, red bar) or treated with fresh TRAIL at 62 ng ml^{-1} (Fresh, green bar), with TRAIL pre-incubated at 37ºC for 24 h (Pre-incubated, blue bar), and with the medium resulting from the 24 h experiment of TRAIL-induced apoptosis (Reused, yellow bar). Cells were imaged for 24 h every 15 min and the fraction of apoptotic cells were calculated by visual inspection of phase contrast images. Error bars are standard deviations obtained by bootstrapping from different images of two biological replicates.

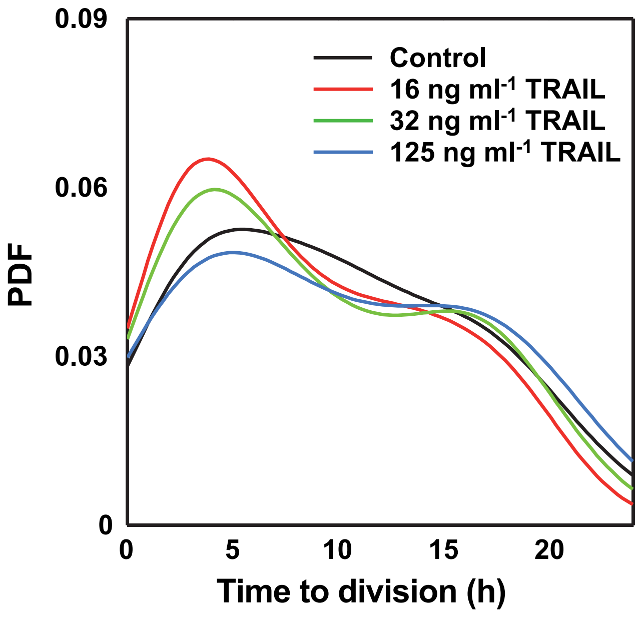

Supplementary Fig. 2. Distributions of cell division times. Probability of time to cell division in clonal and non-synchronized populations of HeLa cells no treated (Control) and treated with different TRAIL doses, obtained by visual inspection of the phase contrast images from 24 h time-lapse. Ensembles of 100-200 cells that divided once during this time-lapse were used to calculate the distributions. For TRAIL-treated samples, only cells surviving TRAIL were considered. Kolmogorov-Smirnov tests between control and TRAIL-treated populations to statistically check for the equality of the distributions could not reject the null hypothesis that control and TRAIL-treated division times came from the same continuous distribution (p-values > 0.3) with 5% significance ('kstest2' in Matlab2014a, Mathworks). We have also measured cell cycle times of 200 recently born HeLa cells by tracking until next division, obtaining an average cell time of 26±7 h (error corresponds to standard deviation).

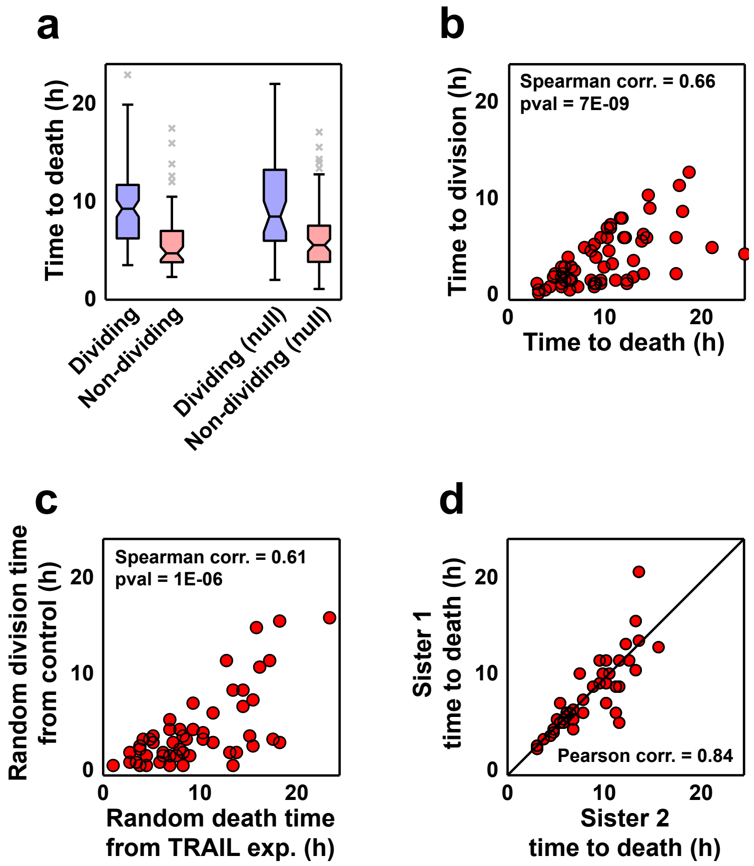

Supplementary Fig. 3. Independence of division and times to death in single cells. **a** Boxplots of times to death for the apoptotic cells undergoing divisions (blue) and not dividing (red) before dying at 16 ng ml^{-1} of TRAIL. The experimental ensemble is shown on the left, while the results of a 'null' simulated ensemble are shown on the right. The 'null' ensemble was generated by randomly sampling division times from the experimental probability distribution in Supplementary Fig. 2 (black line), while times to death were randomly and independently sampled from the experimental distribution shown in Fig. 1c (blue). In the 'null' model, we deemed a cell as 'non-dividing' if its randomly assigned death time was shorter than its assigned division time, and 'dividing' if otherwise. Similar results were obtained with other TRAIL doses (32 and 125 ng ml^{-1}). **b** Correlation between death and division times for cells treated with TRAIL (16 ng ml^{-1}) that divided before dying. In most sibling cells both sisters died within the observation time (70% of apoptotic dividing cells at a TRAIL dose of 16 ng ml^{-1}, 75% at 32 ng ml^{-1} and 86% at 125 ng ml^{-1}). To correlate with division time, we took as death time the average apoptosis time of both sister cells, since they are highly correlated (panel d). **c** Correlation between death and division times for the 'null' simulated ensemble at 16 ng ml^{-1} of TRAIL. **d** Correlation between apoptosis times of sister cell pairs (cells that divided after addition of 16 ng ml^{-1} of TRAIL). For 32 ng ml^{-1} and 125 ng ml^{-1} Pearson's correlation coefficient between apoptosis times of sibling pairs were 0.91 and 0.88 respectively. Boxes cover the range from the lower to the upper quartile of the data. Whiskers indicate maximum and minimum values, excluding outliers which are plotted as individual grey crosses. Horizontal lines inside the boxes represent median values, and notches indicate 95% confidence intervals for the median.

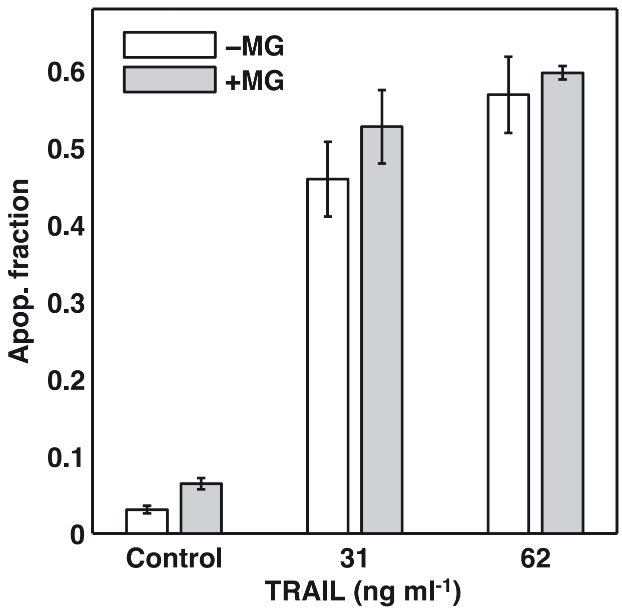

Supplementary Fig. 4. Effect of MitoTracker Green in the fractional killing induced by TRAIL. HeLa cells not stained (-MG, white bars) or stained with MitoTracker Green (+MG, grey bars) were treated with different doses of TRAIL (31 and 62 ng ml^{-1}). Cells were imaged for 24 h every 15 min and the fraction of apoptotic cells were calculated by visual inspection of phase contrast images. Error bars are the standard deviation of six different images (two biological replicates and three different visual fields per replicate).

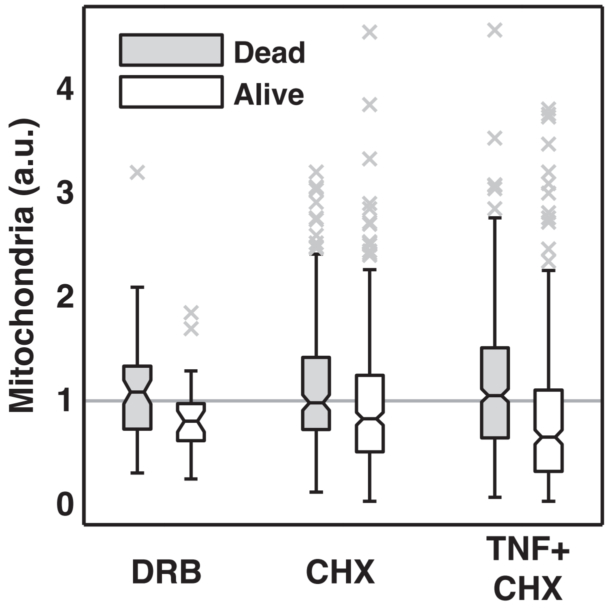

Supplementary Fig. 5. Influence of mitochondrial content on apoptotic cell fate triggered by different inductors. HeLa cells stained with MG were treated with 63 ng ml^{-1} of DRB, 2.5 µg ml^{-1} of cycloheximide (CHX), and a combination of 2.5 µg ml^{-1} of CHX with 20 ng ml^{-1} of TNF. Cells were imaged for 24 h every 15 min. Boxplots of mitochondrial levels of alive (white) and dead (grey) HeLa cells after 24 h of treatment. Mitochondrial values are normalized to average (grey line). Boxes cover the range from the lower to the upper quartile of the data. Whiskers indicate maximum and minimum values, excluding outliers which are plotted as individual grey crosses. Horizontal lines inside the boxes represent median values, and notches indicate 95% confidence intervals for the median. Data are representative of 2 independent experiments.

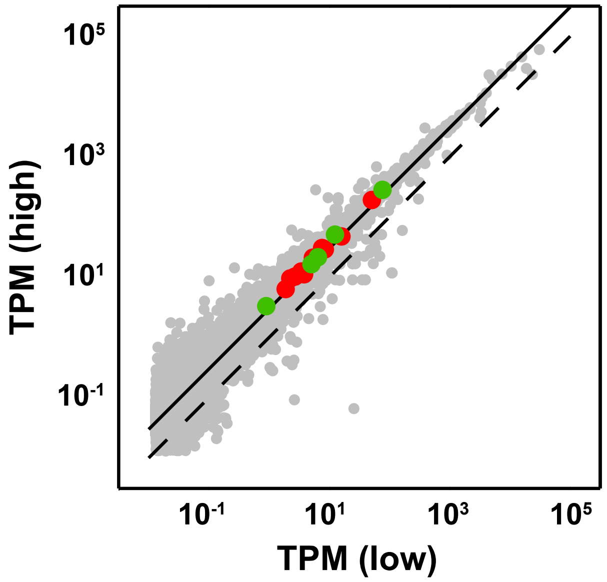

Supplementary Fig. 6. Global scaling of gene expression with mitochondrial content. We sorted a clonal population of HeLa cells in two subpopulations with low and high average mitochondrial mass per cell. These two subpopulations were RNA-sequenced. The expression of each gene given as Transcripts Per Million (TPM) in both subpopulations is represented in a scatter plot (grey dots) in log-log scale. The dashed line corresponds to the line X=Y. The solid line is the regression line to the data and shows a shift due to a global scaling factor of 2.8 for transcript abundance in the subpopulation with high mitochondrial content. The genes coding for the proteins of the apoptotic pathway are shown in red (pro-apoptotic proteins) and green (anti-apoptotic proteins), falling along the global scaling line.

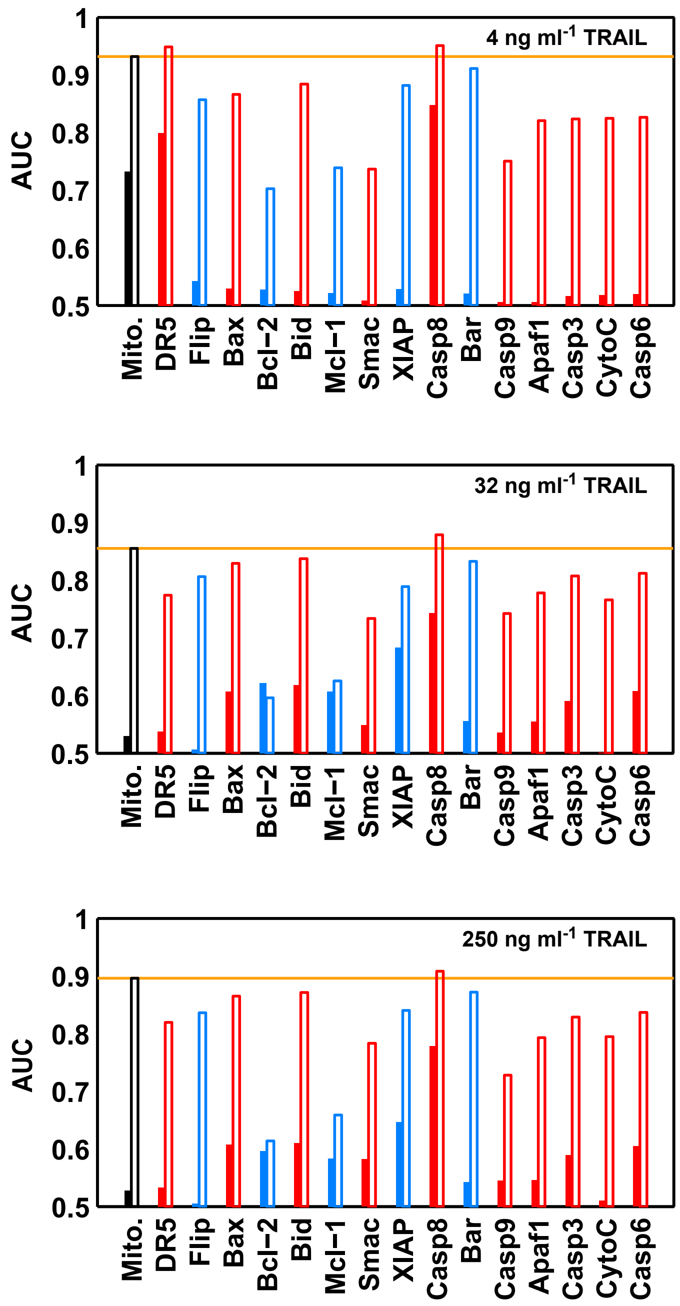

Supplementary Fig. 7. Receptor levels improve discriminatory capacity of mitochondria at low TRAIL doses. Performance of apoptosis fate discrimination using each apoptotic protein as a binary classifier for different TRAIL doses. Red bars correspond to pro-apoptotic proteins and blue bars to anti-apoptotic proteins. Hollow bars: All correlations between mitochondrial mass and apoptotic proteins are included. Filled bars: Only the experimentally observed correlation between mitochondrial mass and DR5 is included. The discriminatory performance of mitochondria is included in orange as a reference. 10,000 cells were simulated at each TRAIL dose.

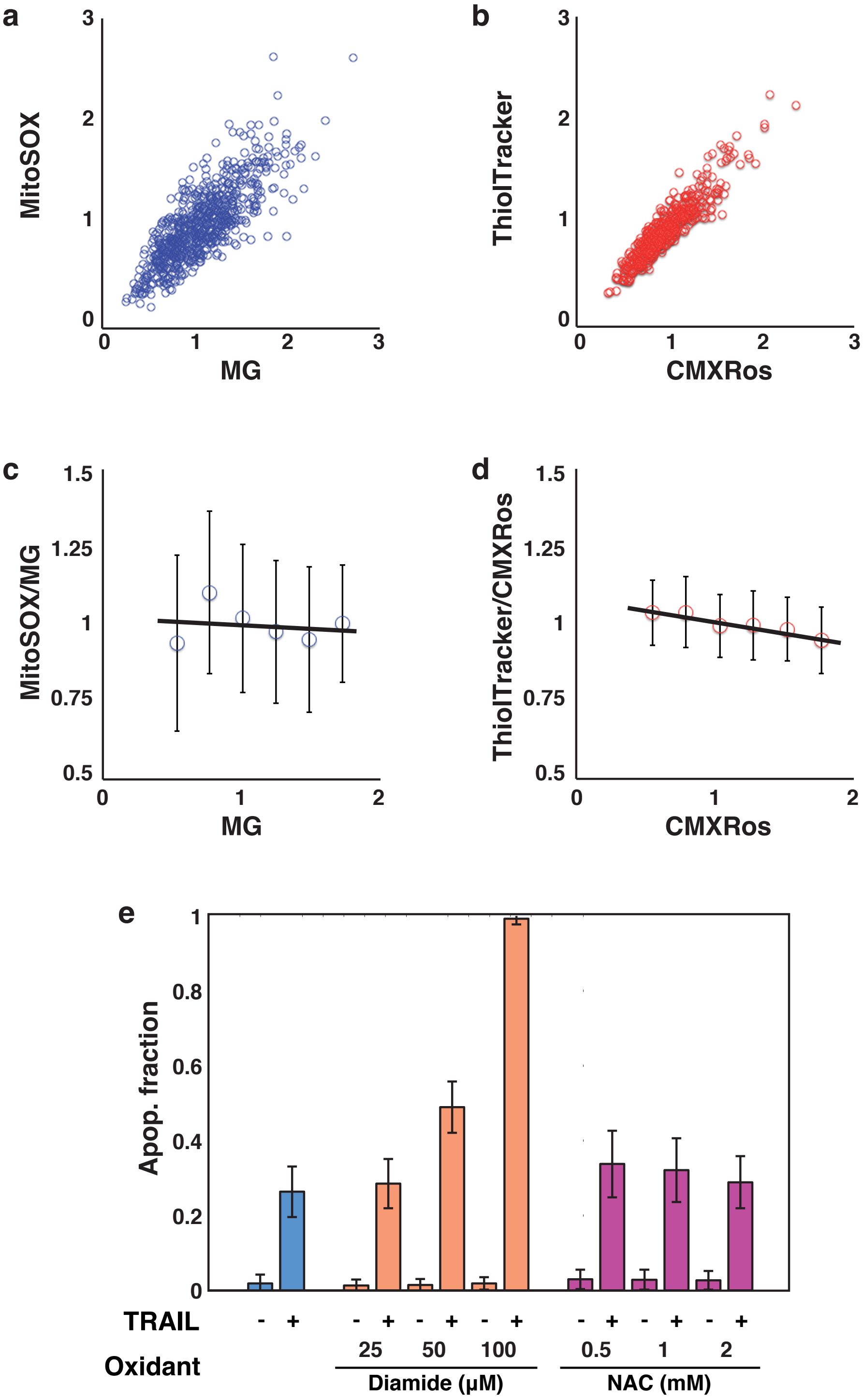

Supplementary Fig. 8. Effect of ROS in TRAIL-induced apoptosis. a ROS production by mitochondria. HeLa cells were co-stained with MG and MitoSOX (a reporter of mitochondrial superoxide) for 2 h. **b** Antioxidant defence of HeLa cells. HeLa cells were co-stained with ThiolTracker (stains reduced thiol group in the cell) and CMXRos. **c** Ratio MitoSOX/MG versus MG. This plot shows not bias in the superoxide production per mitochondria in cells with high mitochondria content versus cells with low mitochondria content. **d** Ratio of ThiolTracker/CMXRos versus CMXRos. This plot shows that the antioxidant defence is proportional to the mitochondria content,

therefore, cells with high mitochondria content have the same oxidative stress than low mitochondria content cells. **e** Effect of ROS in fractional killing induced by TRAIL**.** Apoptotic fraction of HeLa cells after a 2h of pre-treatment with increasing doses of diamide (pro-oxidant, orange) or N-acetyl cysteine (NAC, antioxidant, pink) and a posterior 24 h treatment with TRAIL 31 ng ml^{-1}. Error bars are the standard deviation of six different images (two biological replicates and three different visual fields per replicate).

Supplementary Table 1. Parameters involved in the mitoEARM model.

	Protein	Molecules per cell (population average) [(a)]	Coefficient of variation, CV_p	Logarithmic protein-mitochondria correlation, ρ	Degradation rate, k_{deg} (h^{-1})
1	Ligand	50 × TRAIL [(b)]			0.462
2	Receptor	500	0.36	0.79	0.139
3	Ligand:Receptor	0			0.832
4	Receptor*	0			0.832
5	Flip	2 000	0.29	0.87	0.139
6	Flip:Receptor*	0			0.832
7	Casp8	1 000	0.37	0.86	0.139
8	Casp8:Receptor*	0			0.832
9	Casp8*	0			0.832
10	Bar	1 000	0.36	0.96	0.139
11	Casp8:Bar*	0			0.832
12	Casp3	10 000	0.40 [(c)]	0.77 [(c)]	0.139
13	Casp8*:Casp3	0			0.832
14	Casp3*	0			0.832
15	Casp6	10 000	0.40 [(c)]	0.77 [(c)]	0.139
16	Casp3*:Casp6	0			0,832
17	Casp6*	0			0,832
18	Casp6*:Casp8	0			0,832
19	XIAP	100 000	0.44	0.90	0.139
20	XIAP:Caspase-3*	0			0.832
21	PARP	100 000	0.40 [(c)]	0.77 [(c)]	0.139
22	Casp3*:PARP	0			0.832
23	cPARP	0			0.832
24	Bid	60 000	0.43	0.90	0.139
25	Casp8*:Bid	0			0.832
26	tBid	0			0.832
27	Mcl-1	20 000	0.42	0.60	0.139
28	tBid:Mcl-1	0			0.832
29	Bax	80 000	0.29	0.86	0.139
30	tBid:Bax	0			0.832
31	Bax*	0			0.832
32	$Bax*_m$	0			0.832
33	Bcl-2	30 000	0.40	0.51	0.139
34	$Bax*_m$:Bcl-2	0			0.832
35	$Bax*2_m$	0			0.832

36	$Bax*2_m$:Bcl-2	0			0.832
37	$Bax*4_m$	0			0.832
38	$Bax*4_m$:Bcl-2	0			0.832
39	Pore	500 000	0.55 [(d)]	1.00 [(d)]	0.139
40	$Bax*4_m$:Pore	0			0.832
41	Pore*	0			2.189
42	$CytoC_m$	500 000	0.40 [(c)]	0.77 [(c)]	0.139
43	Pore*:$CytoC_m$	0			0.832
44	$CytoC_r$	0			0.832
45	$Smac_m$	100 000	0.35	0.58	0.139
46	Pore*:$Smac_m$	0			0.832
47	$Smac_r$	0			0.832
48	CytoC	0			0.832
49	Apaf	100 000	0.40 [(c)]	0.77 [(c)]	0.139
50	Apaf:CytoC	0			0.832
51	Apaf*	0			0.832
52	Caspase-9	100 000	0.41	0.59	0.139
53	Apoptosome	0			0.832
54	Apoptosome:Casp3	0			0.832
55	Smac	0			0.832
56	Apoptosome:XIAP	0			0.832
57	Smac:XIAP	0			0.832
58	$Casp3*_{Ub}$	0			0

(a) Values taken from[3], with the only exceptions of *Ligand* and *Receptor*, that have been tuned for best fitting of our data. The values of degradation rates are taken from[5].

(b) In the original model, a dose of 50 ng ml^{-1} of TRAIL was mimicked setting the initial *Ligand* concentration to 3000 molecules, which means 60 molecules per ng ml^{-1} of TRAIL. In our case, we have set 50 *Ligand* molecules per each ng ml^{-1} of TRAIL.

(c) We lack experimental data for some proteins, for which we have assumed a CV of 0.40 and a mitochondrial log-correlation of 0.77. These are averaged values among the proteins for which we do have experiments.

(d) Note that the variable *Pore* is a pseudo-molecular species representing each potential site in the mitochondrial membrane where a pore could be formed. It is directly related to the mitochondrial mass, which is why it has ρ=1. Its coefficient of variation comes from experimental distributions of mitochondria.

Supplementary Table 2. Biochemical reactions and kinetic parameter values used in the mitoEARM model.

	Reaction	***k* *(molec⁻¹ h⁻¹)***	***k_r* *(h^{-1})***	***k_c* *(h^{-1})***
1	$Ligand + Receptor \leftrightarrow [Ligand:Receptor] \rightarrow Ligand + Receptor^*$	1.3e-4	0.0216	216
2	$Receptor^* + Flip \leftrightarrow [Receptor^*:Flip]$	2.16e-2	21.6	
3	$Receptor^* + Casp8 \leftrightarrow [Receptor^*:Casp8] \rightarrow Receptor^* + Casp8^*$	2.16e-3	21.6	21600
4	$Casp8^* + Bar \leftrightarrow [Casp8^*:Bar]$	2.16e-2	21.6	
5	$Casp8^* + Casp3 \leftrightarrow [Casp8^*:Casp3] \rightarrow Casp8^* + Casp3^*$	2.16e-3	21.6	21600
6	$Casp3^* + Casp6 \leftrightarrow [Casp3^*:Casp6] \rightarrow Casp3^* + Casp$	2.16e-3	21.6	21600
7	$Casp6^* + Casp8 \leftrightarrow [Casp6^*:Casp8] \rightarrow Casp6^* + Casp8^*$	2.16e-3	21.6	21600
8	$Casp3^* + XIAP \leftrightarrow [Casp3^*:XIAP] \rightarrow Casp3^*_{Ub} + XIAP$	4.32e-2	21.6	2160
9	$Casp3^* + PARP \leftrightarrow [Casp3^*:PARP] \rightarrow Casp3^* + cPARP$	2.16e-2	21.6	432000
10	$Casp8^* + Bid \leftrightarrow [Casp8^*:Bid] \rightarrow Casp8^* + tBid$	2.16e-3	21.6	21600
11	$tBid + Mcl-1 \leftrightarrow [tBid:Mcl-1]$	2.16e-2	21.6	
12	$tBid + Bax \leftrightarrow [tBid:Bax] \rightarrow tBid + Bax^*$	2.16e-3	21.6	21600
13	$Bax^* \leftrightarrow Bax^*_m$	216	21600	
14	$Bax^*_m + Bcl-2 \leftrightarrow [Bax^*_m:Bcl-2]$	3.09e-1	21.6	
15	$Bax^*_m + Bax^*_m \leftrightarrow Bax^*2_m$	3.09e-1	21.6	
16	$Bax^*2_m + Bcl-2 \leftrightarrow [Bax^*2_m:Bcl-2]$	3.09e-1	21.6	
17	$Bax^*2_m + Bax^*2_m \leftrightarrow Bax^*4_m$	3.09e-1	21.6	
18	$Bax^*4_m + Bcl2 \leftrightarrow [Bax^*4_m:Bcl-2]$	3.09e-1	21.6	
19	$Bax^*4_m + Pore \leftrightarrow [Bax^*4_m:Pore] \rightarrow Pore^*$	3.09e-1	21.6	21600
20	$Pore^* + CytoC_m \leftrightarrow [Pore^*:CytoC_m] \rightarrow Pore^* + CytoC_r$	6.17e-1	21.6	216000
21	$Pore^* + Smac_m \leftrightarrow [Pore^*:Smac_m] \rightarrow Pore^* + Smac_r$	6.17e-1	21.6	216000
22	$CytoC_r \leftrightarrow CytoC$	21600	216	
23	$CytoC + Apaf1 \leftrightarrow [CytoC:Apaf1] \rightarrow CytoC + Apaf1^*$	1.08e-2	21.6	21600
24	$Apaf1^* + Casp9 \leftrightarrow Apoptosome$	1.08e-3	21.6	
25	$Apoptosome + Casp3 \leftrightarrow [Apoptosome:Casp3] \rightarrow Apoptosome + Casp3^*$	1.08e-4	21.6	21600

26	$Smac_r \quad \leftrightarrow \quad Smac$	21600	216
27	$Apoptosome + XIAP \quad \leftrightarrow \quad [Apoptosome\!: XIAP]$	4.32e-2	21.6
28	$Smac + XIAP \quad \leftrightarrow \quad [Smac\!: XIAP]$	1.51e-1	21.6
29	$Receptor^{*} \quad \leftrightarrow \quad Ligand + Receptor$	21.6	0

Supplementary Table 3. Expected correlation between protein pairs due to global mitochondria-protein correlations.

**Protein$_1$**, p_1	**Protein$_2$**, p_2	**Mito. log-correlation of p$_1$**, ρ_{xy}	**Mito. log-correlation of p$_2$**, ρ_{xz}	**Min. p$_1$-p$_2$ log-correlation**, $\rho_{yz}^{(0-)}$	**Max. p$_1$-p$_2$ log-correlation**, $\rho_{yz}^{(0+)}$	**Expected p$_1$-p$_2$ log-correlation**, $\rho_{yz}^{(1/2)}$
Receptor	Flip	0.79	0.87	0.39	0.99	0.69
Receptor	Casp8	0.79	0.86	0.37	0.99	0.58
Casp8	Bar	0.86	0.96	0.68	0.97	0.83
Casp8	Bid	0.86	0.90	0.55	0.99	0.77
Bid	Mcl-1	0.90	0.60	0.19	0.89	0.54
Bid	Bax	0.90	0.86	0.55	0.99	0.77
Bax	Bcl-2	0.86	0.51	0.00	0.88	0.44
XIAP	Smac	0.90	0.58	0.17	0.88	0.52
XIAP	Casp9	0.90	0.59	0.18	0.88	0.53

SUPPLEMENTARY REFERENCES:

1. Albeck JG, Burke JM, Aldridge BB, Zhang M, Lauffenburger DA, Sorger PK. Quantitative analysis of pathways controlling extrinsic apoptosis in single cells. *Molecular cell* **30**, 11-25 (2008).

2. Albeck JG, Burke JM, Spencer SL, Lauffenburger DA, Sorger PK. Modeling a snap-action, variable-delay switch controlling extrinsic cell death. *PLoS biology* **6**, 2831-2852 (2008).

3. Gaudet S, Spencer SL, Chen WW, Sorger PK. Exploring the contextual sensitivity of factors that determine cell-to-cell variability in receptor-mediated apoptosis. *PLoS Comput Biol* **8**, e1002482 (2012).

4. Spencer SL, Gaudet S, Albeck JG, Burke JM, Sorger PK. Non-genetic origins of cell-to-cell variability in TRAIL-induced apoptosis. *Nature* **459**, 428-432 (2009).

5. Bertaux F, Stoma S, Drasdo D, Batt G. Modeling dynamics of cell-to-cell variability in TRAIL-induced apoptosis explains fractional killing and predicts reversible resistance. *PLoS Comput Biol* **10**, e1003893 (2014).

6. Aldridge BB, Gaudet S, Lauffenburger DA, Sorger PK. Lyapunov exponents and phase diagrams reveal multi-factorial control over TRAIL-induced apoptosis. *Mol Syst Biol* **7**, 553 (2011).

7. Stoma S, Donze A, Bertaux F, Maler O, Batt G. STL-based analysis of TRAIL-induced apoptosis challenges the notion of type I/type II cell line classification. *PLoS Comput Biol* **9**, e1003056 (2013).

8. Spencer SL, Sorger PK. Measuring and modeling apoptosis in single cells. *Cell* **144**, 926-939 (2011).

9. Roux J*, et al.* Fractional killing arises from cell-to-cell variability in overcoming a caspase activity threshold. *Mol Syst Biol* **11**, 803 (2015).

10. Zi Z. Sensitivity analysis approaches applied to systems biology models. *IET Syst Biol* **5**, 336-336 (2011).
