# Supplementary material for: Mitochondrial levels determine variability in cell death by modulating apoptotic gene expression
Source: Nat Commun. 2018 Jan 26;9:389. doi: 10.1038/s41467-017-02787-4 (PMC5785974; doi:10.1038/s41467-017-02787-4)
Supplement: Supplementary file 3 — Description of Additional Supplementary Files [file 41467_2017_2787_MOESM3_ESM.docx]

**Description of Additional Supplementary Files**

File Name: Supplementary Software 1

Description: The software contains the matlab codes for the dynamic model of apoptosis use in this paper.
